# One-Step Bark-Like Imitated Polypropylene (PP)/Polycarbonate (PC) Nanofibrous Meltblown Membrane for Efficient Particulate Matter Removal

**DOI:** 10.3390/polym11081307

**Published:** 2019-08-04

**Authors:** Ting-Ting Li, Xixi Cen, Hai-Tao Ren, Fei Sun, Qi Lin, Ching-Wen Lou, Jia-Horng Lin

**Affiliations:** 1 Innovation Platform of Intelligent and Energy-Saving Textiles, School of Textile Science and Engineering, Tianjin Polytechnic University, Tianjin 300387, China; 2 Tianjin and Ministry of Education Key Laboratory for Advanced Textile Composite Materials, Tianjin Polytechnic University, Tianjin 300387, China; 3 Fujian Key Laboratory of Novel Functional Fibers and Materials, Minjiang University, Fuzhou 350108, China; 4 Ocean College, Minjiang University, Fuzhou 350108, China; 5 Fujian Engineering Research Center of New Chinese Lacquer Material, Minjiang University, Fuzhou 350108, China; 6 Department of Bioinformatics and Medical Engineering, Asia University, Taichung 41354, Taiwan; 7 Department of Medical Research, China Medical University Hospital, China Medical University, Taichung 40402, Taiwan; 8 College of Textile and Clothing, Qingdao University, Shandong 266071, China; 9 Laboratory of Fiber Application and Manufacturing, Department of Fiber and Composite Materials, Feng Chia University, Taichung 40724, Taiwan; 10 Department of Fashion Design, Asia University, Taichung 41354, Taiwan

**Keywords:** bark-like imitated, nanofibrous, one-step, meltblown, filtration performance

## Abstract

A bark-like imitated polypr opylene (PP)/polycarbonate (PC) nanofibrous membrane was constructed by one-step meltblown technique for efficient particulate matter (PM) removal. The effects of PC content (0%, 1%, 3%, 5%, and 7%) on membrane thermal stability, microscopic characteristics, filtration performance, hydrophilicity, and water vapor transmission were investigated. The results demonstrated that using facile design of incompatibility and viscosity difference between PC and PP polymers decreases average fiber diameter, creating a bark-like groove appearance and increasing surface potential, making a new PP/PC membrane with high filtration performance. The resultant PP/PC membrane had finer average fiber diameter of 0.63 μm, which was nearly 89.41% lower than PP membranes (5.95 μm), and its quality factor (0.036 Pa^−1^) was nearly 2.12 times than that of PP membranes (0.017 Pa^−1^) with the die hole diameter of 0.5 mm. This fabrication technique of a special meltblown filter membrane saves the cost of die retrofitting and post-processing, which provides an innovative method for particulate efficient removal of high efficient filters.

## 1. Introduction

Environmental pollution problems seriously endanger human health. In particular, particulate matter (PM) with aerodynamic diameters of less than 2.5 μm in atmosphere are one of the most serious types of air pollution [1,2,3]. PM can carry toxic and harmful substances, such as polycyclic aromatic hydrocarbons and heavy metals, and can easily enter human bronchioles and alveoli and cause respiratory infections, cardiovascular diseases, pneumoconiosis, cancer, and other diseases [4,5,6,7,8]. Generally, the treatment strategies of air pollution exist in two forms: Source treatment and terminal filtration. The source treatment strategy is effective, and includes reducing vehicle exhaust emissions and managing polluting enterprises. However, this strategy is difficult to implement, costly, and requires a substantial amount of time [9]. The development of terminal high-efficiency air filters that can effectively reduce the damaging effects of PM on human health is urgently required.

Recently, a number of different methods became available for the fabrication of filter, of which meltblowing has emerged as the preferred technique for enhanced particulate filtration. The meltblown filter is a leader in advanced filtration materials with high specific surface area, high yield, high strength, fine fiber diameter, and narrow pore size distribution [10]. However, the minimum average fiber diameter of a typical meltblown filter is 1–2 μm, resulting in a gap between it and the electrospun fiber diameter in the range of 100–500 nm, which limits it wider application in high-efficiency air filters. Thus, refining the diameter of the meltblown filter has become an urgent problem. Many researchers have used different methods to refine the meltblown fiber. For example, in 2019, Deng et al. prepared a multi-scale micro/nano fiber membrane by adding polystyrene (PS) to polypropylene (PP) during the meltblowing process, and the average fiber diameters of the crude and fine fibers were about 8 and 1 μm, respectively [11]. In 2019, Zhang et al. fabricated nonwoven micro/nano fibers with a branch structure by adding polyethylene glycol (PEG) to polypropylene (PP) during the meltblowing process, and showed that the microfibers can be as high as 10 μm in diameter and the fine fibers can be as small as 200 nm [12]. In 2018, Soltani et al. mixed water-soluble polymer, sulfonated poly(ethylene terephthalate) (SP), hydrophilic polybutylene terephthalate (PBT), and hydrophobic polyvinylidene fluoride (PVDF) meltblown, and then washed away SP post-treatment to produce a fiber diameter of 200 nm [13]. In 2013, Uppal et al. prepared a filter with a fiber diameter of 520–590 nm using modular meltblown molding technology [14]. In 2012, Hassan et al. spun a meltblown filter with a fiber diameter of 300–1500 nm by retrofiting the die [15]. In 2013, Lalagiri et al. prepared a meltblown air filter with fiber diameter of 520–2100 nm through nano-die holes [16]. In 2007, Ellison et al. produced a poly(butylene terephthalate) (PBT), polypropylene (PP), and polystyrene (PS) meltblown membrane with average fiber diameters less than 500 nm by a single orifice meltblowing apparatus [17]. In 2004, Zhang et al. combined AQ polymer with PP to prepare a two-component meltblown filter and split the fiber to obtain fine fibers [18]. Although the above studies significantly improve the fiber diameter of the meltblown filter, these methods, especially die retrofitting and post-processing, are complex and greatly increase production cost. 

**Figure 1:**
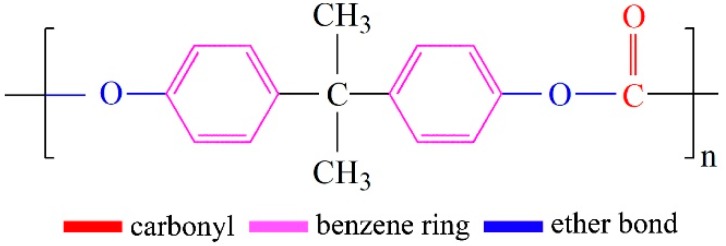
Chemical structural formula of the polycarbonate.

Polypropylene (PP) is a thermoplastic resin polymerized from propylene. It has low density (0.89–0.91 g/cm^3^), easy processing, low cost, and excellent physical properties, so it has become the main material for air filters [19]. However, the electrostatic properties of PP are relatively low. Polycarbonate (PC) is a transparent amorphous thermoplastic resin, and its structural formula is shown in Figure 1. The presence of polar group carbonyl in the PC molecular chain provides high insulation and dielectric constant (3.0–3.2) for materials [20,21]. 

Herein, a special bark-like imitated nanofiber membrane was fabricated by the incompatibility and viscosity difference between PP and PC using one-step meltblown technology. Under the action of hot air at the fiber formation site, the PC filler with high viscosity acts as a dispersed phase, which draws the low viscosity PP substrate to the nanofiber scale. In this work, the relationship between different PC content and the microscopic characteristics, fiber diameter and fiber surface morphology, filtration performance, surface potential, water contact angle, and water vapor transmission of the nanofibrous membrane were comprehensively explored. In addition, the mechanism between the fiber structure and filtration performance of the membrane was clarified. The bark-like imitated PP/PC nanofiber meltblown membrane will exhibit a promising application in air filtration.

## 2. Experimental Section

### 2.1. Materials

Polypropylene (PP) resin with a melt flow rate (MFR) of 35 g/10 min was purchased from Dongguan Xiangsheng Plastic Co., Ltd. (Guangdong, China) and dried at 80 °C for 2 h prior to usage. Polycarbonate (PC) resin with a MFR of 14.4 g/10 min was purchased from SABIC Innovative Plastics (China) Co., Ltd. (Guangzhou, China) and dried at 120 °C for 4 h before use. 

### 2.2. Preparation of the PP/PC Masterbatches

PP/PC blending masterbatches were prepared by a single screw granulator (Qingdao Keshengda Plastic Machinery Co., Ltd., China, SJ45X36). The processing parameters are listed in Table 1 and the as-prepared masterbatches were named PP, PC-1, PC-3, PC-5, and PC-7 according to different content of the PC (Table S1, Supporting Information). 

### 2.3. Preparation of the PP/PC Membranes

**Figure 2:**
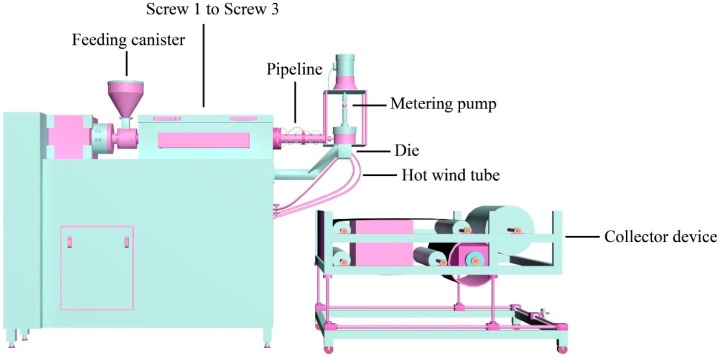
Schematic drawing of the meltblown process.

PP/PC membranes were prepared by a single screw meltblown apparatus (Tianjin Shengruiyuan Machinery Technology, China). The name of the as-prepared membranes corresponds to the blending masterbatches (Table S1, Supporting Information). To begin, the blending masterbatches were dried in a blast drying oven (Tianjin Tongda Experimental Electric Furnace Plant, China, YC-02) at 80 °C for 4 h. Then, the masterbatches were melted and pushed forward by the shearing force of the extruder. Afterwards, the molten polymer was sequentially passed through the pipeline, metering pump and die. Finally, under the action of the hot air in the hot wind tube, the polymer was blown from the die holes to the collection web. The density of die is 9 holes/cm and the diameter of die holes is 0.5 mm. Figure 2 and Table 2 respectively show the schematic illustration of the preparation process and the processing conditions for the blending membranes.

### 2.4. Characterization

The thermal stability information of the as-prepared membranes was obtained through a thermogravimetric analyzer (Neichi Scientific Instruments Trading (Shanghai) Co., Ltd., China, TG-209F3). The samples were heated from 50 to 800 °C in nitrogen atmosphere at 10 °C min^−1^. The compatibility and thermal behavior were studied via a differential scanning calorimetry (Neichi Scientific Instruments Trading (Shanghai) Co., Ltd.), China, DSC-200F3) in nitrogen atmosphere. The samples were first heated from 30 to 300 °C at 20 °C·min^−^^1^ and maintained at 300 °C for 5 min, then cooled to 30 °C at 10 °C·min^−^^1^ and finally heated to 300 °C at 10 °C·min^−1^ for the second melting. The crystallinity of the PP/PC membranes was calculated by the Equation (1) [22,23,24]:

(1) $$X_{C}\%=\frac{\Delta H_{m2}}{(1-\varphi )\;\Delta H_{m2}^{0}}\times 100$$

where Δ*H*_*m*2_ is the second heat of fusion measured by the sample, *φ* is the weight fraction of the PC additive, and the Δ$H_{m2}^{0}$ is the heat of fusion of the 100% crystalline PP (Δ$H_{m2}^{0}$ = 209 J/g) [24].

The viscosity of the polymers was measured by a capillary rheometer (Jilin Huabo Science and Technology Industry Co., Ltd., China, HMLB-400), and the temperature was set according to the temperature of the metering pump of the meltblown (260 °C).

The specific surface area of samples was measured with an automatic adsorption desorption analyzer (Autosorb-iQ2-MP, Conta Instrument Co., Ltd., Boynton Beach, FL, USA). Before adsorption testing, samples (0.3–0.4 g) were degassed by nitrogen (N_2_) blowing at 100 °C for 5 h for the removal of vapor and impurities.

### 2.5. Fiber Diameter Measurements

The microscopic characteristics of the as-prepared membranes were obtained through the scanning electron microscope (Phenom-World Company, Eindhoven, Holland, Phenom pure) and the field emission scanning electron microscope (HITACHI S4800, HITACHI Company, City, Japan,) using acceleration voltages of 5 and 10 kV, respectively. Before characterization, the samples were sputter coated with gold for 120 s. Fiber diameter was measured using Image-Pro software. For each membrane, 300 individual fiber diameters were measured and averaged.

### 2.6. Fiber Surface Roughness Measurements

The surface roughness of the membrane fiber was measured using a true color confocal microscope (Axio CSM700, Karl Zeiss Company, Heidenheim, Germany,) with 20× objective lens, 120 s scanning speed, and 40 nm resolution. For each sample, five measurements were taken and averaged.

### 2.7. Surface Potential Measurements

The surface potential of the membranes was measured through a vibrating capacitive electrometer (EST-102, Beijing Huajinghui Technology Co., Ltd., China). The higher surface potential results in the more excellent PM filtration performance source by the electrostatic force. The measurement distance between probe and sample was 2 cm, and 10 measurements were taken and averaged.

### 2.8. Filtration Efficiency Measurements

The filtration efficiency and pressure drop of membranes were examined using an automated filter tester (TOPAS AFC-131, TOPAS GmbH Company, Dresden, Germany). The filtration efficiency was measured using dioctyl sebacate (DEHS) aerosol wth the range of 0.208–4.595 μm at a flow rate of 3.4 m^3^·h^−1^. The pressure drop was measured at five different flow rates (0.7, 1.4, 2, 2.7, and 3.4 m^3^·h^−1^). The testing area of each sample was 176.71 cm^2^_._ The filtration efficiency and pressure drop were calculated by the Equations (2) and (3), respectively [25,26]:

(2) $$E=1-(\frac{C_{down}}{C_{up}})$$

where *E* is the particulate filtration efficiency, and *C_down_* and *C_up_* are the upstream and downstream aerosol concentrations, respectively.

(3) $$\Delta P=P_{1}-P_{2}$$

where Δ*P* is the pressure drop, *P*_1_ is the pressure before filtration, and *P*_2_ is the pressure after filtration.

### 2.9. Water Contact Angle and Water Vapor Transmission Measurements

A water contact angle (CA) measuring instrument (JC2000DM, Shanghai Zhongchen Digital Technology Equipment Co., Ltd., China) was employed to measure the water contact angle. Approximately 5 μL of deionized water was used per test and each sample was measured at least five times. The water vapor transmission of membranes was measured with a water vapor transmission meter (YG(B)216, Wenzhou Darong Textile Instruments Co., Ltd., China), according to the desiccant method in ASTM E 96. The test temperature and humidity were 38 ± 1 °C and 90% ± 2%, respectively. The rate of water vapor transmission (WVT) was calculated from the Equation (4):

(4) $$WVT(g\cdot {\left(m^{2}\cdot h\right)}^{-1})=\frac{\Delta m-\Delta m^{\prime }}{A\cdot t}$$

where Δ*m* (g) is the difference between the two weights of the same test dish assembly of the sample, Δ*m′* (g) is the difference between the two weights of the same test dish assembly of the blank sample, *A* (m^2^) is the effective test area, which is 0.00283 m^2^, and *t* (h) is the test time.

## 3. Results and Discussions

### 3.1. Microscopic Characteristics of Meltblown Membranes

**Figure 3:**
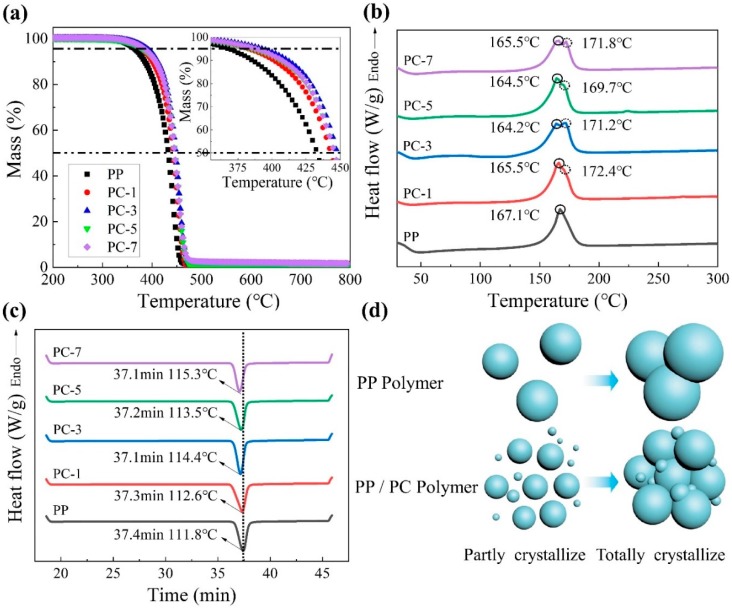
(**a**) Thermogravimetric (TG) thermograms; differential scanning calorimetry (DSC) thermograms; (**b**) First heating; (**c**) cooling; and (**d**) the nucleation processes of polypropylene (PP)/polycarbonate (PC) meltblown membranes.

**Figure 4:**
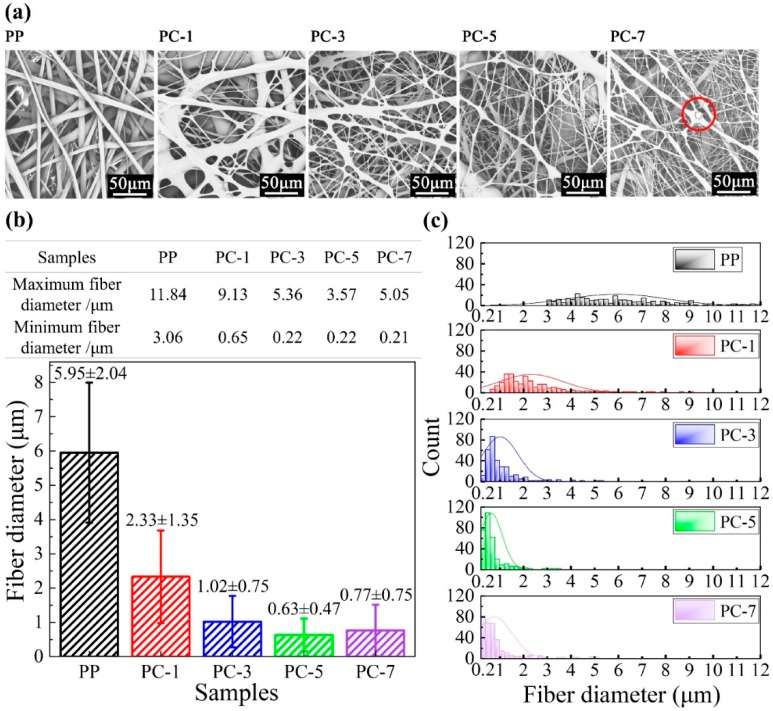
(**a**) SEM images; (**b**) average fiber diameters (the maximum and minimum fiber diameters are shown in the table); and (**c**) diameter distribution of PP, PC-1, PC-3, PC-5, and PC-7 fibers.

The TG thermogram (Figure 3a) reflects the degree of thermal degradation of PP/PC meltblown membranes. The curve change was observed clearly by setting the temperature range between 200 and 700 °C. As shown in Figure 4 and Table S2 (Supplementary Information), the addition of PC significantly increased the thermal degradation temperature of the PP membranes, thereby improving the thermal stability of the membranes. PC-3 had the best thermal stability compared to pure PP sample, and its temperatures of 5% and 50% mass decomposition increased by 6.76% and 3.42%, respectively.

DSC thermogram (Figure 3b,c) was carried out to shed light on the thermal behavior of PP/PC meltblown membranes. It can be observed that the thermal behavior of membranes varies with PC content. Figure 3b shows the first heating curve of the PP/PC membranes. Compared with the pure PP membranes (167.1 °C), a new melting peak appeared after PC was introduced, and the difference after PC content exceeded 1% was more obvious. The potential reason behind this trend is that PP is a nonpolar crystalline polymer and PC is a polar amorphous polymer. Different structures result in poor compatibility between PP and PC, resulting in the appearance of a double peak phenomenon [27]. Remarkably, the appearance of the right peak is maybe attributed to the PC because the melting point of PC (250 °C) is higher than PP (169 °C). This phenomenon matches the results of TG thermogram and convincingly verifies the existence of PC on the PP/PC membranes.

In the crystallinity (*X*_c_, Table 3) calculated by Equation (1), the crystallinity of PP membranes containing PC was improved. In particular, PC-3 has the highest crystallinity (48.76%), which is more than 1.12 times than that of pure PP membranes (43.67%), and increased by nearly 11.66%. Combined with Figure 3c, the crystallization peak and the heating rate of the sample containing PC increased. This phenomenon indicates that PC can act as nucleating agent for PP. Pure PP generates crystal nucleus by molecular motion, the nucleation rate is slow and produces large grains. However, after adding PC, the crystallization behavior of PP changed, and PP homogeneous nucleation was transformed into heterogeneous nucleation, and grain size was refined. In turn, the amount of space charge captured by the crystal-amorphous boundary of the fine crystals increased, and thus the charge trapping capacity, charge density, and charge stability of the membranes were enhanced. the crystallization process is shown in Figure 3d [28]. 

### 3.2. Fiber Diameter

**Figure 5:**
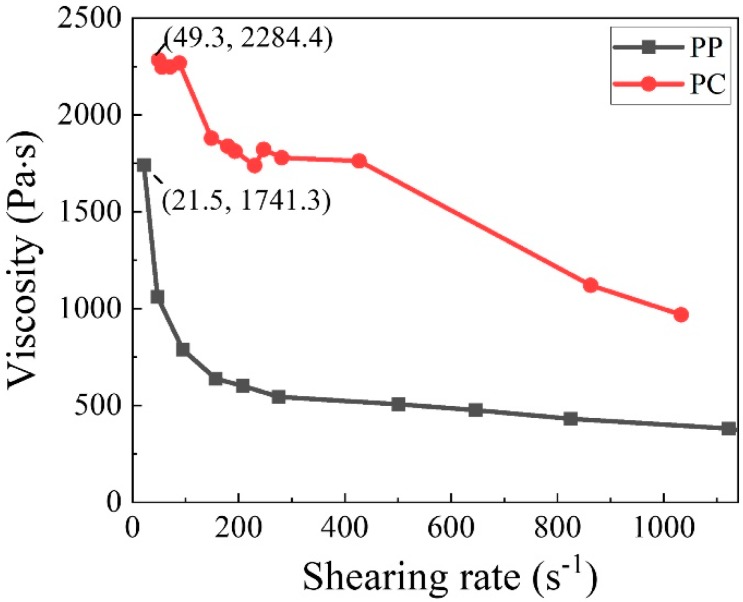
The viscosity curves of PP and PC.

**Figure 6:**
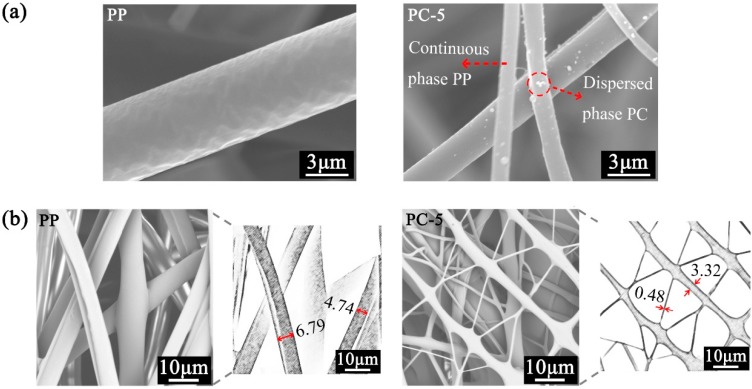
PP and PC-5 (**a**) fiber surface and (**b**) SEM original image at 4500× magnification, as well as the pictures after they were denoised by Photoshop.

**Figure 7:**
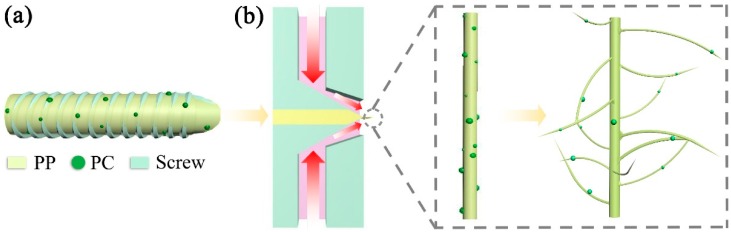
The pull-out process of PC to PP: PP and PC under the action of (**a**) high temperature screw shear force and (**b**) hot air.

Figure 4 shows the fiber diameter distribution of PP membranes with different PC content. The SEM image of the membranes (Figure 4a) shows that the increase in PC content has a great influence on fiber structure. The fiber diameter is dramatically decreased when PC content increases from 1% (PC-1) to 7% (PC-7). Combination with Figure 4b,c, it can be observed that the degree of the refinement of the fiber diameter was not positively correlated with PC content. The smallest average fiber diameter (0.63 μm) was obtained at PC-5, which is nearly 89.41% lower than PP (5.95 μm) under the same process conditions. However, as the PC content continues to increase, the fiber diameter shows an upward trend. The reason behind this is the PC gradually becomes self-adhesive and forms a continuous phase, eventually forming liquid drops in the membranes when the PC content exceed 5%. The liquid drop is indicated by the red circle of Figure 4a. Although the production of the PC-7 liquid drops causes the average fiber diameter to rise, the refining strength was not inferior to PC-5 and the finest fiber diameter reached 210 nm, as shown in Figure 4b. The refinement mechanism mainly stems from the incompatibility and the viscosity difference between PP and PC [29]. The viscosity curves of PP and PC are shown in Figure 5. PP is a linear chain hydrocarbon polymer and a nonpolar crystalline material, and PC is a polar amorphous polymer with an ester group (–COO–) in its molecular chain. The different structures result in lower interface forces and compatibility, and thus cannot be formed by the thermodynamically compatible homogeneous system [30,31]. Meanwhile, the high content of PP and the low content of PC respectively become continuous phase and dispersed phase (Figure 6a) in the meltblown process. The result for the above reason is that PC has a “pull-out” effect on PP. To further show the “pull-out” effect, SEM images were zoomed to 4500× and denoised it with Photoshop (Figure 6b). From these figures, we can see that the high-viscosity PC filler pulls the PP substrate to both sides of the fiber at the fiber-forming site of the die holes under the action of hot air, and then draws the PP fiber to the nanoscale. The detailed formation principle schematic of “pull-out” effect was shown in Figure 7.

The fiber diameter range and the fiber proportion of each scale can be observed from Figure 4c. The span is inversely proportional to fiber uniformity, and high uniformity contributes to improved filtration efficiency of membranes [32]. As shown in Figure 4c, as the PC content increases, the fiber uniformity increases remarkably, but after the PC content exceeds 5%, it shows a downward trend, mainly due to the generation of liquid drops.

### 3.3. Fiber Surface Morphology

**Figure 8:**
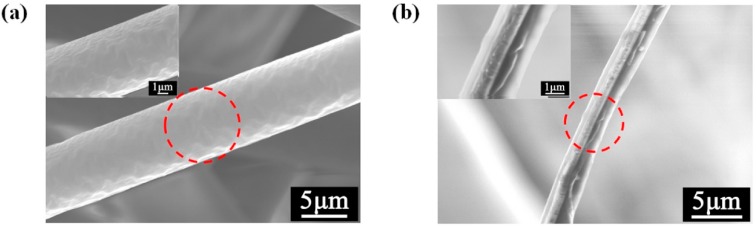
SEM images of (**a**) PP and (**b**) PC-5 meltblown membranes.

The microscopic surface of the fiber at 18,000× magnification was observed with a field emission scanning electron microscope and is shown in Figure 7. The “pull-out” effect of PC on PP changed the smooth fiber surface of PP (Figure 8a) and produced a bark groove with a certain depth (Figure 8b). The special groove structure not only increased the specific surface area of the membranes and effectively increased the contact area between the PM and the fiber, but also facilitated PM interception, which is of great significance to the improvement of filtration performance [33]. 

**Figure 9:**
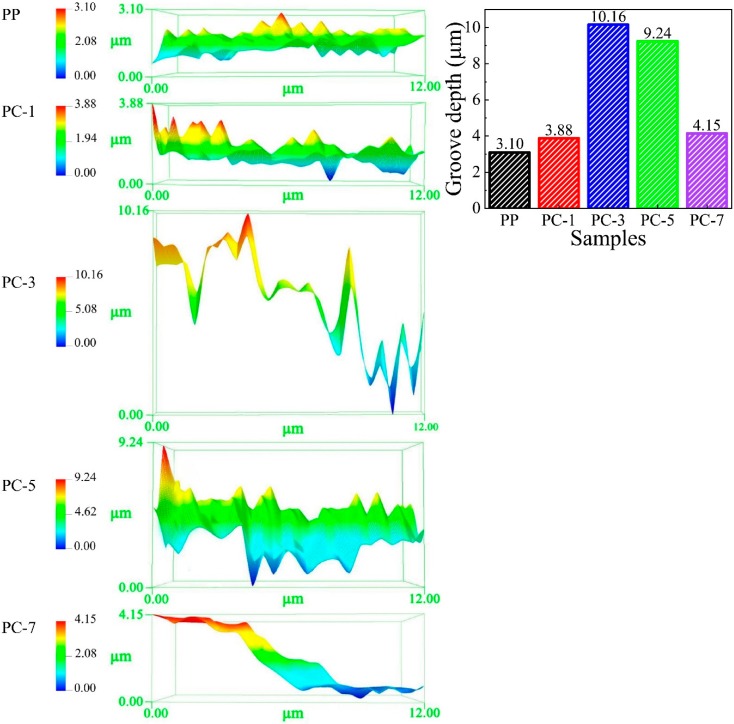
3D surface morphology and groove depth of PP, PC-1, PC-3, PC-5, and PC-7.

The fiber surface roughness test was performed to analyze the influence of different PC content on fiber structure. The test results are listed in Table 4. The roughness of the fiber surface was significantly increased after the addition of PC, and first increased and then decreased with the increase of PC content [34]. The highest roughness (1.03 μm) was obtained at PC-3, which is more than 5.15 times than that of pure PP fiber (0.2 μm), and improved by nearly 415%. However, when the PC content exceeded 3%, the PC gradually changed from the dispersed phase to the continuous phase and the “pull-out” effect on the PP and the roughness were decreased, that is, the groove became shallow. This test verified that the PP fibers containing PC had grooves on the surface. Figure 9 shows the 3D surface morphology and the groove depth of the PP fibers with different PC content, and it can be found that PC content greatly influenced the surface morphology of PP/PC fiber. Pure PP fiber had the flattest surface, in which the distance from the high point to the low point was only 4.15 μm. Surface undulation increased after the introduction of PC, especially when PC-3 reached 10.16 μm, which was 2.45 times that of pure PP fiber. When the PC content exceeded 3%, the surface of the fiber flattened. The results shown in the 3D morphology match the data in Table 3, further verifying that the addition of PC can cause grooves on the surfaces of PP fibers.

### 3.4. Surface Potential of Membranes

**Figure 10:**
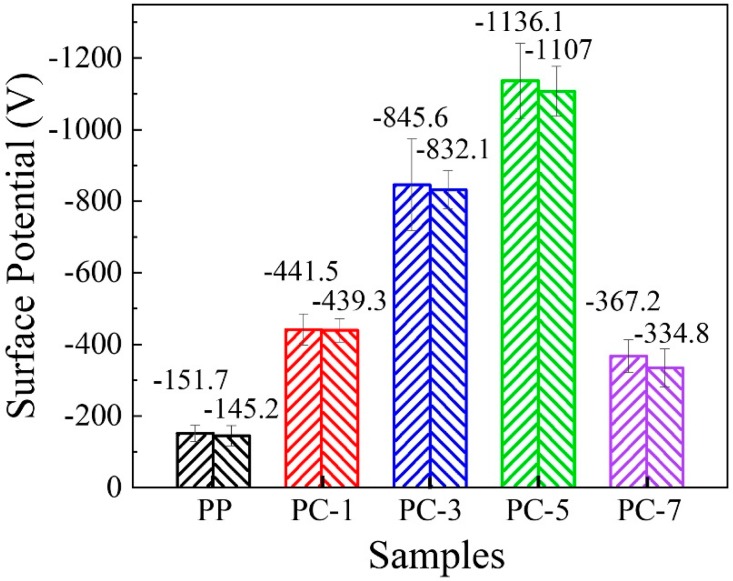
Surface potential of the PP/PC meltblown membranes after initial and indoor storage for 30 days.

The surface potentials of the samples were tested to study the effect of PC content on the electrostatic properties of the PP/PC membranes. As shown in Figure 10, the addition of PC significantly improved the surface potential of the PP membranes, namely increased the surface charge amount [35]. The surface potential of the pure PP membranes was only −151.7 V, whereas that of PC-5 reached −1136.1 V, which is nearly 7.49 times higher. In combination with the average fiber diameter (Figure 4), it can be observed that the increase in surface potential is consistent with the trend of fiber diameter of PP/PC membranes, that is, the surface potential increased with PC content and decreased after the content exceeded 5%. The reason behind this can be attributed to the following reasons: The first aspect is that PC is an electret material, and thus have high dielectric constant and excellent insulation properties, resulting in an increase in charge retention. The second aspect is that the PC improves the crystallinity of PP and refines the grain size, resulting in crystal-amorphous interfaces and enhancing the capturing of space charge. The third aspect is that the “pull-out” effect of PC on PP fiber increases the density of the fiber web structure and the specific surface area of the fiber, which helps to capture and store the charged particles to increase the surface electrostatic potential of the membrane. When the PC content exceeded 5%, it showed a decreasing trend, which was mainly due to the decrease in fiber uniformity caused by the increase in liquid drops. Consequently, the efficiency of the ultrafine fiber that increases the charge amount was reduced. To evaluate the effect of PC on charge decay or charge storage ability of membrane, the PP/PC membranes were stranded indoor for 30 days (temperature: 15 °C, humidity: 40%). As shown in Figure 10, the surface potentials of the five kinds of meltblown samples after one month of storage had no considerable changes, and PC-1 had the most excellent charge stability. These phenomena clearly show that the addition of PC significantly elevates the charge amount of the PP membranes and enhances the stability of the stored charge.

### 3.5. Filtration Efficiency of Membranes

**Figure 11:**
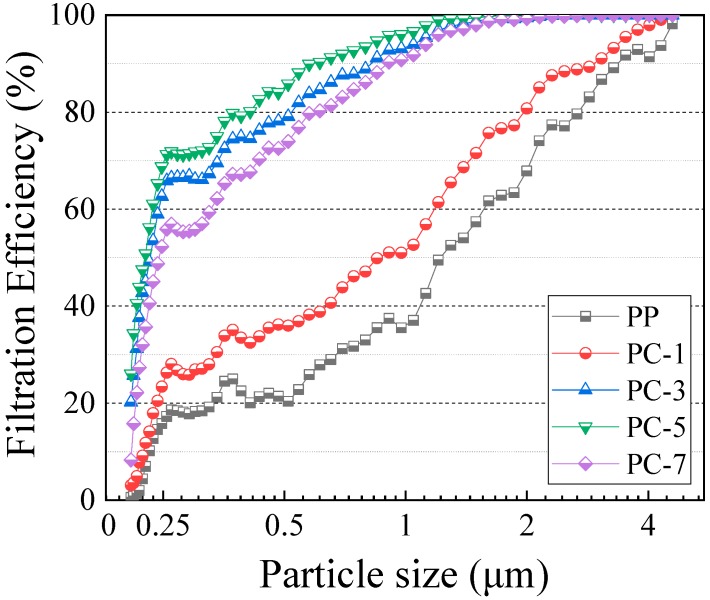
Filtration efficiency of DEHS (0.208–4.595 μm) of PP meltblown membranes with different PC content at 3.4 m^3^/h flow rate.

Filtration efficiency and pressure drop are very important values in the measurement of the filtration performance of filter. A filter with high filtration efficiency easily intercepts PM in the atmosphere, but the corresponding pressure drop value is high, that is, the breathing resistance is large. Therefore, the balancing filtration efficiency with pressure drop is the key in the study of filters. Gravity sedimentation, inertial collision, Brownian diffusion, and electrostatic adsorption mainly occur in filtration mechanisms [36]. The filtration effect of the first three is mainly determined by the structure of the filter, such as fiber diameter, filter thickness, and basis weight, and the electrostatic adsorption is related to the amount of charge on the filter surface [37]. Generally, decreasing the average fiber diameter and increasing the amount of surface charge of the filter can elevate the filtration efficiency. Figure 11 shows the filtration efficiency of PP/PC meltblown membranes at different DEHS particle sizes (0.208 to 4.595 μm) with a test flow rate of 3.4 m^3^/h. The basis weights of the membranes are listed in Table S3. Combined with Table 5, it can be found that the filtration efficiency of PC-5 was 71.9%, which is 53.9% of that of pure PP membranes, when the particle size of the PM was 0.3 μm. Additionally, when the particle size reached 1.858 μm, the filtration efficiency of PC-5 reached 100%, whereas that of the PP membranes was only 62.2%, which increased by nearly 60.77%. When the particle size was 2.478 μm, the filtration efficiency of PC-3 also reached 100%, whereas the filtration efficiency of PP membranes remained low (77.7%). When the particle size reached 4.303 μm, the membranes containing PC reached 100%, whereas the pure PP membranes were only 90.8%. The experimental results show that the addition of PC significantly improved the filtration efficiency of the PP membranes. The filtration efficiency first increased and then decreased with the increase of PC content, and PC-5 had the most excellent filtration efficiency, followed by PC-3.

**Figure 12:**
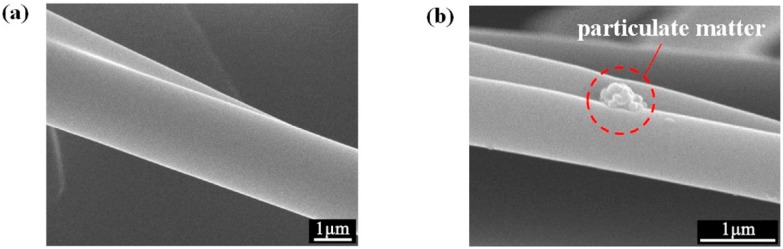
SEM images of PC-5 (**a**) before and (**b**) after 20 times of DEHS aerosol filtration test at membrane fiber surface groove.

The reason behind this trend is potentially attributed to two aspects: Filter structure and surface charge content. On the one hand, PC has the effect of “pull-out” on PP fiber, and this effect reduces the average fiber diameter (Figure 4b), improves fiber uniformity (Figure 4c), and increases specific surface area (Table 6) of the filter. Decrease in the fiber diameter reduces the pore size among the fibers to help intercept the PM [16]. Furthermore, increase in specific surface area results in the increase of fiber surface energy to adsorb more PM. The membranes containing PC were fluffy and had bark-like grooves. This special structure not only broadened the filtration channel, but also intercepted some particles into the groove (Figure 12). On the other hand, PC, acting as a nucleating agent for PP, increased the crystallization temperature, crystallization rate, and crystallinity of the membranes, resulting in an enhance in the surface potential of the filter (Figure 10) to adsorb more PM through the electrostatic power [38].

**Figure 13:**
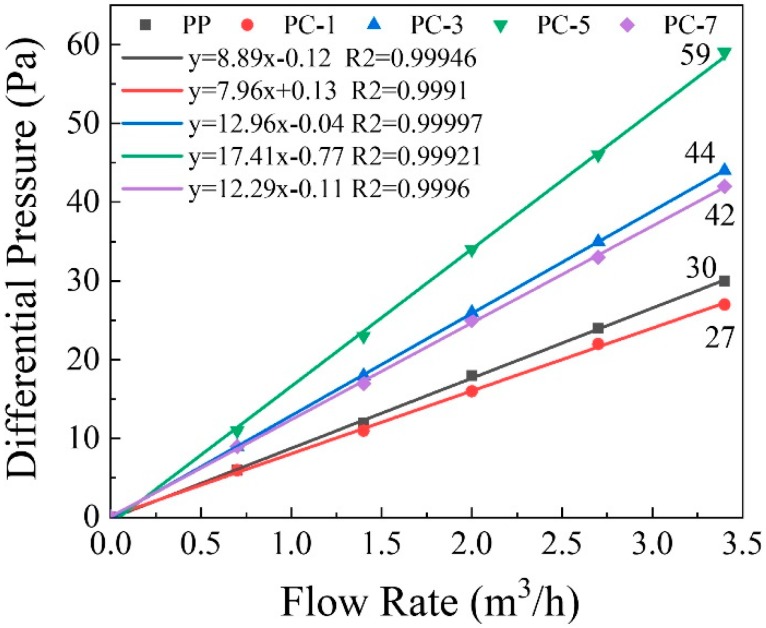
Pressure drop of the PP/PC membranes at five different flow rates (0.7, 1.4, 2, 2.7, and 3.4 m^3^/h).

**Figure 14:**
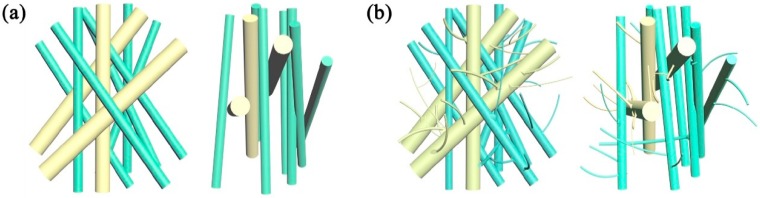
Main and left view schematic diagram of (**a**) PP and (**b**) PC-1.

Pressure drop is an important parameter in the study of air filters. Increasing the pressure drop enhances the respiratory resistance, which seriously affects the comfort of the human body when wearing a mask. As shown in Figure 13, the correlation between the pressure drop of the membranes and the air flow rate is strong, and pressure drop increases with flow rate. At a flow rate of 3.4 m^3^/h, the pressure drop of PC-1 was smaller than that of pure PP meltblown membranes, which was only 27 Pa. The main reason behind this phenomenon is that a small portion of the PP fibers became finer due to by the “pull-out” effect of PC, while most of the crude fibers still broaden the respiratory channels. The schematic illustration is shown in Figure 14. As more PC was introduced, the “pull-out” effect enhanced, and most of the fibers showed a fine fiber state, thus the pressure drop increased. The highest pressure drop was obtained at PC-5 (59 Pa). When the PC content was 7%, the pressure drop decreased, mainly due to the PC from the dispersed phase which gradually shifted to a continuous phase and formed large liquid drops during the meltblown process. 

To evaluate the overall filtration performance, the useful criterion is the quality factor (*Q*_f_). The filtration quality factor is defined as Equation (5) [35,39].

(5) $$Q_{f}=\frac{-ln(1-E)}{\Delta P}$$

where *E* and Δ*P*, respectively, are the filtration efficiency and pressure drop of the filter, which are obtained from the filtration tester.

**Figure 15:**
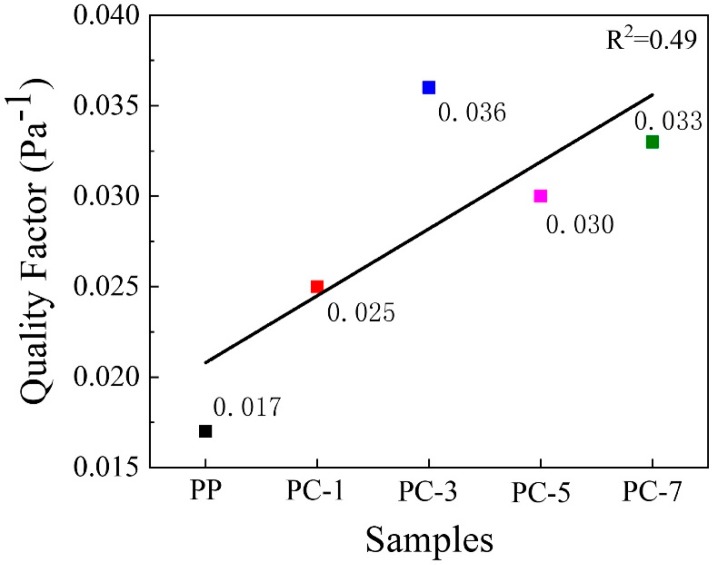
The quality factor of PP meltblown membranes containing different content of PC.

Figure 15 compares the filtration quality factors of PP meltblown membranes with different PC content. The increase in the quality factor was not positively correlated with the PC content but increased first and decreased after the content reached 5%, as shown in the linear fitting. PC-3 had the highest quality factor of 0.036 Pa^−1^, which was 2.12 times than that of pure PP membranes (0.017 Pa^−1^). However, although PC-5 exhibited the most excellent filtration performance, its quality factor was lower than PC-3 due to its high pressure drop. Compared with other meltblown filters, PP membranes containing PC had great filtration performance. For example, the quality factor of commercial meltblown filter was 0.033 Pa^−1^. In 2019, Zhang et al. produced micro-nano meltblown filter by changing the content of PEG in PP and the temperature of the die, and the best quality factor was 0.026 Pa^−1^ [12]. In 2018, Zhang et al. prepared a high filtration efficiency electret PP meltblown filter by adjusting the charging voltage and charging distance, and die they used had a hole diameter of 0.25 mm and the highest quality factor (0.025 Pa^−1^) [40].

### 3.6. Water Contact Angle and Water Vapor Transmission of Membranes

**Figure 16:**
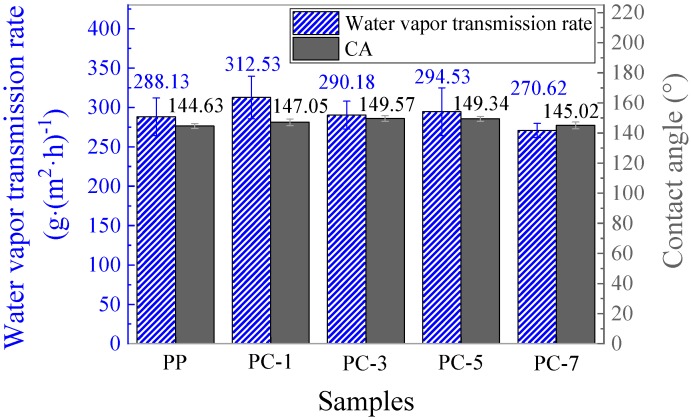
Moisture permeability and water contact angle of the PP meltblown membranes with different PC content.

**Figure 17:**
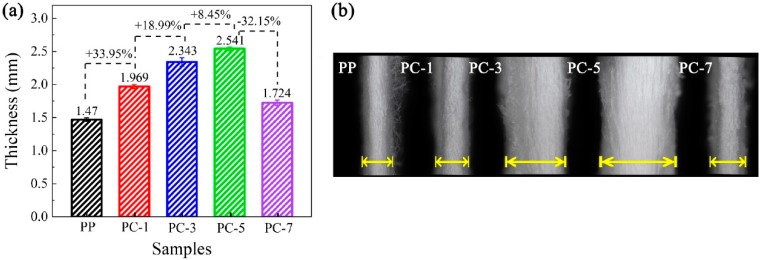
(**a**) Thickness and (**b**) cross-sectional physical pictures of PP meltblown membranes with different PC content.

The water vapor transmission is closely related to the comfort performance of a mask. When the water vapor transmission of the filter is good, water vapor generated from the breath is transported to the outer surface of the filter, and the breathing area remains dry and bacterial growth is prevented. As shown in Figure 16, the water vapor transmission rate of the membranes was enhanced after the addition of PC, and the contact angle (CA) was improved as well. Due to the incompatibility between PP and PC, the adhesion of the fiber during fiber formation was reduced, and thus the membrane tended to be fluffy, as shown in Figure 17. The unique fluffy structure broadened the water vapor transmission channels and improved the water vapor dissipation speed. The most excellent water vapor transmission (312.53 g·(m^2^·h)^−1^) was obtained at PC-1, which was nearly 8.47% higher than pure PP membranes. PC-3 had the highest water CA (149.57°), which was 4.94° higher than pure PP membranes. However, when the PC exceeded 5%, the water vapor transmission and the CA dropped sharply because of the generation of liquid drops.

## 4. Conclusions

In this study, nanofiber membranes based on PP and PC were fabricated by one-step meltblown technique for high filtration performance. Compare to other techniques that produces nanofiber meltblown membranes, this technique does not need equipment retrofit and the process is simple. Profiting from the incompatibility and viscosity difference of two materials, the PC has the “pull-out effect” on PP to prepare the nanoscale bark-like fiber. Under the same process conditions, the smallest average fiber diameter (0.63 μm) was obtained at PC-5, which is nearly 9.44 times lower than PP fiber (5.95 μm), and the finest fiber diameter of PC-7 can reach 210 nm. Meanwhile, the special fiber surface structure of bark-like groove reduces the specific surface area of the fiber, facilitates the interception of PM in the groove, and improves the filtration performance of the membranes. PC-5 has the highest initial surface potential of −1136.1 V due to PC as a nucleating agent for PP, which is nearly 7.49 times higher than the −151.7 V of pure PP. In addition, the water vapor transmission of the membranes is essential to the comfort performance of the mask. The addition of PC can increase the water vapor transmission rate and the fluffiness of the PP membranes, resulting in accelerated water vapor transmission and retained the dryness of the breathing area. Lastly, PC fillers can effectively improve the filtration efficiency and reduce the pressure drop of the PP meltblown membranes. In detail, PC-3 has the best filtration performance, as its quality factor is 0.036 Pa^−1^, which is 2.12 times and 1.09 times than that of pure PP (0.017 Pa^−1^) and commercial meltblown membranes (0.033 Pa^−1^), respectively. It is therefore concluded that the bark-like imitated PP/PC nanofiber meltblown membrane has potential application for enhanced air particulate filtration.

## Figures and Tables

**Table 1:** Processing parameters of blending masterbatches.

Parameter	Screw 1	Screw 2	Screw 3	Nozzle	Screw Speed/r·min^−1^	Pelletizin Speed/r·min^−1^
**Temperature/°C**	240	260	275	200	21	32

**Table 2:** Processing conditions of meltblown membranes.

**Screw 1/°C**	**Screw 2/°C**	**Screw 3/°C**	**Pipeline/°C**	**Metering Pump/°C**	**Die/°C**
220	275	290	270	260	252
**Air/°C**	**Metering Pump Flow** **/mL·min^−1^**	**Air Pressure** **/MPa**	**Collector Speed** **/cm·min^−1^**	**Winding Sticks Speed** **/cm·min^−1^**	**Distance** **/cm**
PP: 200 PC-1, PC-3, PC-5, PC-7: 158	50	0.035	44	22	20

**Table 3:** Thermal properties of the membranes.

Samples	First Heating		Cooling		Second Heating		
	*T*_m1_ /°C	Δ*H*_m1_ /J·g^−1^	*T*_c_ /°C	Δ*H*_c_ /J·g^−1^	*T*_m2_ /°C	Δ*H*_m2_ /J·g^−1^	*X*_c_ /%
PP	167.1^1^	87.56	111.8	101.9	161.0	91.27	43.67
PC-1	165.5^1^, 172.4^2^	89.38	112.6	105.4	166.6	96.53	46.65
PC-3	164.2^1^, 171.2^2^	95.55	114.4	107.1	159.2	98.85	48.76
PC-5	164.5^1^, 169.7^2^	89.24	113.5	97.94	158.2	93.2	46.94
PC-7	165.5^1^, 171.8^2^	84.76	115.3	92.51	160	92	47.33

^1^ The melting peak of PP; ^2^ The melting peak of PC.

**Table 4:** Roughness parameters of the investigated samples (*R*_a_–average roughness, *R*_q_–root mean square).

Samples	Ra/μm ± SD	Rq/μm ± SD
PP	0.20 ± 0.08	0.22 ± 0.08
PC-1	0.30 ± 0.06	0.34± 0.07
PC-3	1.03 ± 0.14	1.25 ± 0.15
PC-5	0.46 ± 0.36	0.53 ± 0.44
PC-7	0.31 ± 0.02	0.35 ± 0.02

**Table 5:** Filtration efficiency (%) of the meltblown samples to DEHS with primary particle size of 0.208–4.595 μm at 3.4 m^3^/h flow rate.

Particle Size/μm	PP	PC-1	PC-3	PC-5	PC-7
0.208	0.5	3.3	20.8	25.5	8.2
0.248	15.1	22.7	63.1	68.8	51.7
0.3	18.0	26.3	66.1	71.9	55.7
0.372	26.0	35.2	75.0	80.1	67.6
0.612	28.1	39.3	84.0	90.7	80.9
0.909	37.6	52.8	92.9	95.9	90.5
1.858	62.2	75.0	99.3	100.0	99.0
2.478	77.7	90.0	100.0	100.0	99.7
2.856	85.5	88.8	100.0	100.0	100.0
4.303	90.8	100.0	100.0	100.0	100.0
4.595	99.4	100.0	100.0	100.0	100.0

**Table 6:** Specific surface areas of the samples.

Samples	PP	PC-1	PC-3	PC-5	PC-7
**S_BET_/m^2^·g^−1^**	4.151 ± 0.08	5.404 ± 0.05	9.218 ± 0.07	7.801 ± 0.08	6.312 ± 0.10

## Supplementary Materials

The following are available online at https://www.mdpi.com/2073-4360/11/8/1307/s1, Table S1: The compositions of blending masterbatches and meltblown membranes, Table S2: The thermal degradation temperature of membranes, Table S3: The basis weights of membranes.
