# Supplementary material for: One-Step Bark-Like Imitated Polypropylene (PP)/Polycarbonate (PC) Nanofibrous Meltblown Membrane for Efficient Particulate Matter Removal
Source: Polymers (Basel). 2019 Aug 4;11(8):1307. doi: 10.3390/polym11081307 (PMC6723958; doi:10.3390/polym11081307)
Supplement: Supplementary file 1 [file polymers-11-01307-s001.pdf]

Supplementary Materials

Table S1. The compositions of blending masterbatches and meltblown membranes.

Masterbatches and meltblown membranes	PP/Kg	PC/Kg	Mass ratio of PP:PC /%
PP	5	0	0
PC-1	4.95	0.05	99:1
PC-3	4.85	0.15	97:3
PC-5	4.75	0.25	95:5
PC-7	4.65	0.35	93:7

Table S2. The thermal degradation temperature of membranes.

Samples	The temperature of 95% mass/℃	The temperature of 90% mass/℃	The temperature of 50% mass/℃
PP	368.4	385.7	433.1
PC-1	382.6	401.2	443.1
PC-3	393.3	411.6	447.9
PC-5	385.0	406.0	445.9
PC-7	388.1	407.5	445.8

Table S3. The basis weights of membranes.

Samples	PP	PC-1	PC-3	PC-5	PC-7
Average/g · cm^{-2}	0.0207	0.0231	0.0233	0.0235	0.0236
Standard deviation	0.048	0.043	0.007	0.024	0.003
